# Glutamatergic alterations in the cortex of genetic absence epilepsy rats

**DOI:** 10.1186/1471-2202-8-69

**Published:** 2007-08-29

**Authors:** Monique Touret, Sandrine Parrot, Luc Denoroy, Marie-Françoise Belin, Marianne Didier-Bazes

**Affiliations:** 1 INSERM, U842, Lyon; Université de Lyon, Lyon1, Faculté de Médecine Laennec, UMR-S842, Lyon, F-69372, France; 2 Neurochem, Université de Lyon, Lyon1, Faculté de Pharmacie, Lyon 1 France; 3 CNRS FRE 3006, Lyon; Université de Lyon, Lyon1, Faculté de Pharmacie, Lyon 1 France

## Abstract

**Background:**

In absence epilepsy, the neuronal hyper-excitation and hyper-synchronization, which induce spike and wave discharges in a cortico-thalamic loop are suspected to be due to an imbalance between GABA and glutamate (GLU) neurotransmission. In order to elucidate the role played by GLU in disease outcome, we measured cortical and thalamic extracellular levels of GLU and GABA. We used an *in vivo *quantitative microdialysis approach (no-net-flux method) in an animal model of absence epilepsy (GAERS). In addition, by infusing labelled glutamate through the microdialysis probe, we studied *in vivo *glutamate uptake in the cortex and thalamus in GAERS and non-epileptic control (NEC) rats. Expression of the vesicular glutamate transporters VGLUT1 and VGLUT2 and a synaptic component, synaptophysin, was also measured.

**Results:**

Although extracellular concentrations of GABA and GLU in the cortex and thalamus were not significantly different between GAERS and NEC rats, cortical GLU uptake was significantly decreased in unrestrained awake GAERS. Expression of VGLUT2 and synaptophysin was increased in the cortex of GAERS compared to NEC rats, but no changes were observed in the thalamus.

**Conclusion:**

The specific decrease in GLU uptake in the cortex of GAERS linked to synaptic changes suggests impairment of the glutamatergic terminal network. These data support the idea that a change in glutamatergic neurotransmission in the cortex could contribute to hyperexcitability in absence epilepsy.

## Background

Generalized absence seizures, mainly seen in childhood, are characterized by loss of consciousness associated with bursts of bilaterally synchronous spike and wave discharges (SWDs). The Genetic Absence Epilepsy Rat from Strasbourg (GAERS) [1,2] is recognized as a valid tool for investigating the human disorder. These rats develop spontaneous absence seizures at about postnatal day 40 that persist throughout life. As in the human disease, the SWDs in GAERS are generated in a cortico-thalamic loop involving reciprocal glutamatergic projections and gamma amino butyric acid (GABA) inter-neurons [2]. Several electrophysiological and pharmacological studies have suggested the involvement of GABA and glutamate (GLU) in the initiation and spreading of SWDs [2], with a predominant role of GABA. Although no obvious anatomical differences in these two systems have been reported in GAERS compared to non-epileptic control (NEC) rats, increased extracellular GABA levels in the thalamus and cortex [3] and reduced GABA uptake in thalamic synaptosomes [4] have been reported in GAERS. As regards the glutamatergic system, we previously observed deregulation of the metabolism of GLU and its membrane transporters in the cortex and/or thalamus of young GAERS before the occurrence of seizures [5,6], suggesting an early impairment of the GLU neuro-circuitry, which may be involved in the genesis of the pathology. In adult rats, electrophysiological studies have demonstrated an increased response to NMDA and non-NMDA glutamatergic receptor activation in the cortex of GAERS compared to NEC rats [7], which may play a major role in the control of absence seizures in the thalamus [8]. Furthermore, we, and others found deregulation of GLU metabolism [9,5] and perturbation of membrane transporter gene expression [6] in the cortex. However, the *in vivo *consequences of these perturbations on basal levels of GLU and its clearance from the extracellular space are not known.

In order to understand the involvement of GLU in the genesis of SWDs, we measured basal extracellular GLU and GABA levels in GAERS and NEC rats. We also evaluated GLU transport capacity using an *in vivo *microdialysis approach in awake animals. Furthermore, we investigated possible alterations in glutamatergic terminals in the GAERS cortex and thalamus by studying the expression of the neuronal GLU vesicular transporters VGLUT1 and VGLUT2 [10], and of synaptophysin, one of the most abundant proteins in synaptic vesicles, used as a marker of terminals [11,12].

## Results

### Microdialysis experiments

The microdialysate values (C_dial_) obtained when no exogenous GLU or GABA was perfused through the probe did not differ between NEC rats and GAERS in either the thalamus or cortex (Table 1). The true extracellular concentrations (C_ext_), which reflect *in vivo *release and uptake processes, were also determined using the no-net flux method. In the two structures studied no difference between NEC rats and GAERS was seen for GLU and GABA (Table 1). However, a difference in the variance (F37.1, p = 0.041) of GLU values have been observed in the cortex between GAERS, and NEC, suggesting a possible instability of excitation only present in the cortex of epileptic rats.

**Table 1:** Comparison of extracellular levels (C_ext_, i.e. C_in _= C_out_), microdialysate concentrations (C_dial_, i.e. C_in _= 0 mol/L), and in vivo extraction efficiency (Ed) i.e. slope of C_in_-C_out _vs C_in_) of GLU and GABA determined by the "no net flux" method (for details, see Methods) in absence epilepsy rats (GAERS strain) and non-epileptic Wistar (NEC) rats

	**GLU (μmol/L)**		**GABA (nmol/L)**	
	GAERS	NEC	GAERS	NEC

**Cortex**	(5)	(5)	(5)	(5)
C_ext_	1.67 ± 0.44	0.91 ± 0.01	18.1 ± 8.1	16 ± 6.1
C_dial_	0.78 ± 0.10	0.68 ± 0.1	10 ± 3.0	12.5 ± 4.2
Ed	0.59 ± 0.18	0.76 ± 0.10	0.67 ± 0.12	0.82 ± 0.05
**Thalamus**	(5)	(6)	(5)	(6)
C_ext_	3.31 ± 146	4.82 ± 1.90	18.5 ± 4.4	14.1 ± 1.9
C_dial_	1.94 ± 0.78	2.26 ± 0.96	15.0 ± 3.3	12.5 ± 2.4
Ed	0.63 ± 0.1	0.47 ± 0.08	0.83 ± 0.05	0.87 ± 0.09

Number of rats per group in brackets

### Estimation of GLU uptake using exogenous radioactive GLU

**Figure 1:**
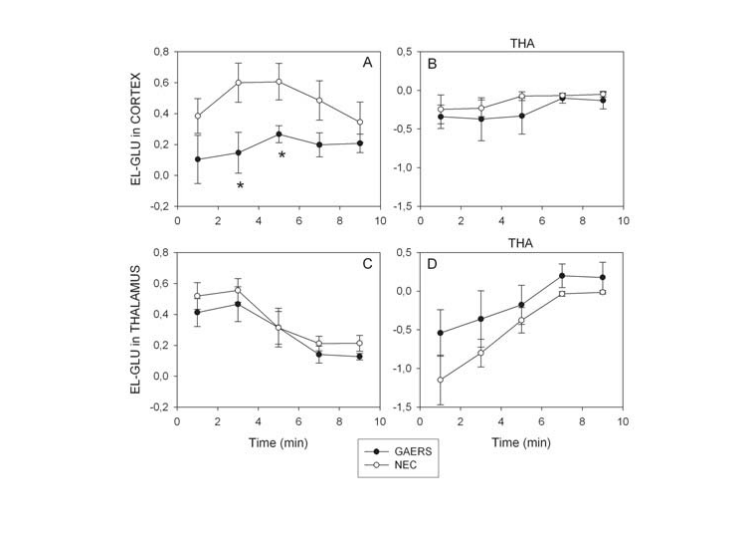
Uptake of exogenous radioactive GLU in the cortex of GAERS (n = 6) and NEC rats (n = 6) and thalamus of GAERS (n = 6) and NEC rats (n = 6). 250 μM ^3^H-GLU and 120 μM ^14^C-MAN were infused for 10 min with or without 20 mM THA and microdialysis samples collected every 2 min. The results, expressed as the mean ± SEM, are plotted as the extraction fraction E_L-GLU_, versus time

In the cortex, GLU uptake was lower in GAERS than in NEC rats (Student's t test, *p < 0.05) when a mixed solution of labeled MAN and GLU was perfused (Figure 1A). In the presence of 20 mmol/L THA, a GLU uptake blocker, GLU uptake was reduced and the difference between the two strains disappeared (Figure 1B). When the same experiment was carried out in the thalamus, no difference was seen between GAERS and NEC rats either in the presence or absence of uptake inhibitor (Figure 1C,D).

### Western blot analysis

#### VGLUT1 expression

**Figure 2:**
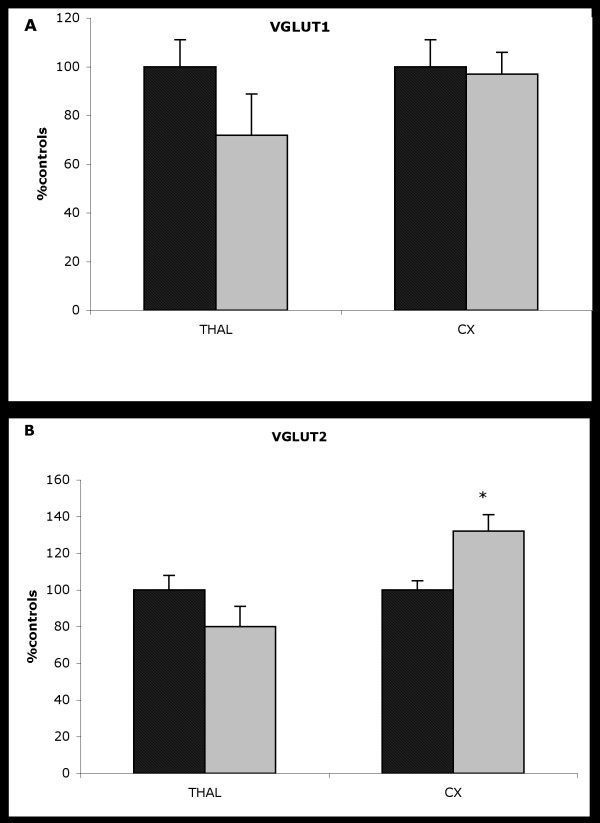
Expression of vesicular GLU transporter proteins in the cortex and thalamus of adult GAERS (grey bars) and NEC controls (hatched bars). Homogenates were subjected to SDS-PAGE and immunoblotted with anti-VGLUT1 (A) or anti-VGLUT2 (B) antibodies. The relative quantification was performed using a fluorimager. The results are represented as a percentage of the value in NEC rats (mean ± SEM; n = 6). Values were compared to control rat values with a two-tailed Student's t test (*p < 0.05)

Quantification of the immunolabeled band at the expected molecular weight of about 59 kDa revealed no difference in protein levels in either the thalamus or cortex between GAERS and NEC rats (Figure 2).

#### VGLUT2 expression

When the immunolabeled band at the expected molecular weight of about 62 kDa was quantified, an increase in protein levels (37% P < 0.01) was seen in the cortex, but not the in thalamus, in GAERS compared to NEC rats (Figure 2).

#### Synaptophysin expression

**Figure 3:**
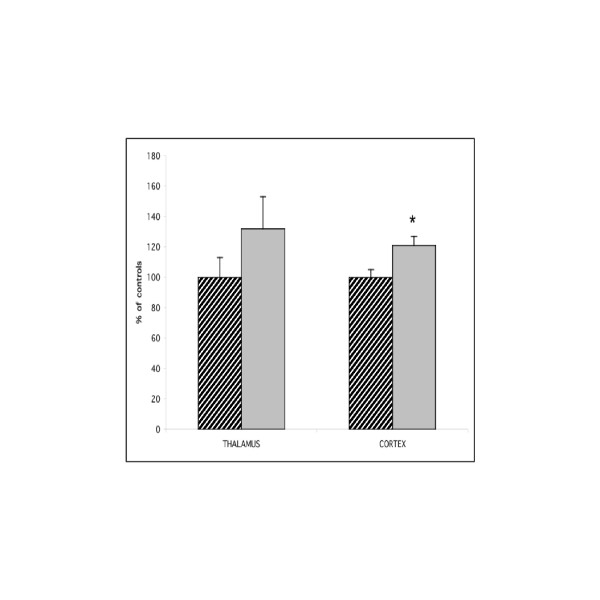
Expression of synaptophysin protein in cortex and thalamus of adult GAERS (grey bars) compared to controls (NEC) (hatched bars). Sample homogenates were subjected to SDS-PAGE and immuno-blotted with anti-synaptophysin antibodies. The relative quantification was performed by a fluorimager. The results are represented as a mean percentage of control rats ± SEM. GAERS values (n = 6) were compared to control rat values (n = 6) with a two-tailed Student's T test (*p < 0,05)

When the immunolabeled band found at the expected molecular weight of about 38 kDa was quantified, a significant increase (21%, p < 0.05) was seen in the cortex, but not in the thalamus, in GAERS compared to NEC rats (Figure 3).

## Discussion and conclusion

The identification of the biochemical and anatomical targets impaired in absence epilepsy is crucial to the understanding of the genesis of the disease and for focusing therapy. The present work provides biochemical data suggesting an alteration in GLU transmission, restricted to the cortex of GAERS. These data are in accordance with previous studies demonstrating: a) a primary role of corticothalamic neurons in the synchronized excitation of thalamic relay and reticular neurons in GAERS [13], b) initiation of the paroxysmal oscillation by a cortical focus in WAG/Rij rats, another model of absence epilepsy [14], and, c) immediate cessation of SWD on applying the anti-absence drug, ethosuximide, to a focalized zone in the cortex, but not the thalamus, in GAERS [15].

Using an *in vivo *approach, we demonstrated decreased GLU uptake in the cortex of unrestrained awake adult GAERS. In contrast, quantitative *in vivo *microdialysis failed to show any significant change in basal GABA or GLU extracellular levels in GAERS in the two structures involved in SWD (thalamus and cortex). However, the variances for the GLU values were larger in the cortex of GAERS, but not in the thalamus, suggesting, an instability in glutamate excitation restricted to the cortex in epileptic rats. However, SWDs were present in some of the time intervals in which the microdialysis samples were collected, but not in others, which might increase the variability of the GLU. Further studies taking into account this experimental aspect by performing sub-minute sampling during brain microdialysis will be of interest to more precisely describe alterations in extracellular GLU in the cortex of GAERS.

The lower *in vivo *GLU uptake in the cortex of GAERS may be due to an impairment of GLU transporters in astrocytes. These cells are a major contributor to the clearance of extracellular GLU via two main glial transporters, GLAST and GLT1, which are situated in the vicinity of the synaptic cleft and play a crucial physiological role in the normal functioning of neurons [16,17]. In a previous study [6], we found decreased levels of GLAST mRNA in the cortex of adult GAERS, without a similar change in protein expression. This could reflect impairment of the transcription and/or turnover of GLAST protein, leading to the lower level of GLU uptake seen in the present study by in vivo microdialysis. From a physiological point of view, this lack of GLU reuptake could favor spillover of the neurotransmitter outside the synaptic cleft and this could be involved in the neuronal synchronization and firing amplification giving rise to SWDs.

The second main finding of the present work was increased VGLUT2 expression in the cortex of adult GAERS, but not in the thalamus, while VGLUT1 expression appeared normal. Interestingly in normal adult rat brain, VGLUT2 protein expression is mainly restricted to lamina IV of the neo-cortex, while its mRNA is found in the thalamus [10,18,19]. In contrast, VGLUT1 is expressed in all layers [20] and probably originates from cortico-cortical projections, as pyramidal neurons express VGLUT1 mRNA [21]. Thus, the increased VGLUT2 expression in the cortex of GAERS strengthens the idea of a glutamatergic alteration in the cortex and suggests that this may involve a glutamatergic thalamocortical input. Finally, since VGLUT2 levels are high at birth and rapidly reach adult values [22,23], one may hypothesize that the higher VGLUT2 expression seen in the cortex in adult GAERS, together with the higher levels of synaptophysin, a marker of synaptic plasticity, might correspond to a developmental process that leads to functional synaptic alterations. However, further studies to determine changes in VGLUT expression during the development of GAERS, especially in young animals before the onset of SWD, are needed to explore this hypothesis. Such work will be of interest given the important role of GLU in the regulation of CNS development [24], especially in the induction or elimination of synapses and synapse plasticity [25,26], and the developmental cortical morphological alterations described in the WAG/Rij epilepsy model [27].

Together, these observations of alterations in the cortical glutamatergic system in adult GAERS linked to impairment of cortical astrocytic function in terms of GLU reuptake and metabolism are in line with the cortical focus theory of absence epilepsy.

## Methods

### Animals

Male Wistar rats from the Genetic Absence Epilepsy Rats from Strasbourg (GAERS) strain and non-epileptic control (NEC) Wistars were used in this study. GAERS display recurrent generalized absence seizures characterized by bilateral and synchronous SWDs on the EEG, concomitant with behavioral arrest. The animals were kept and used in experiments according to the European Communities Council Directives of November 24, 1986 (86/609/EEC). For microdialysis studies, the rats (250–300 g) were anaesthetized with chloral hydrate (400 mg/kg/i.p.) and placed in a stereotaxic frame (David Kopf, USA) with the body temperature maintained close to 37.5°C using a heated under-blanket (Harvard Instruments, USA). The skull was exposed and, after drilling appropriate holes, a cannula-guide (CMA12, CMA, Sweden) was implanted either i) in the cortex at a 30° angle (coordinates relative to the bregma: AP -3.3 mm, L +2.3 mm, V -2.5 mm below the brain surface) or ii) in the ventrobasal thalamus (coordinates relative to the bregma: AP -3.5 mm, L +2 mm, V -6 mm below the brain surface) according to the atlas of Paxinos & Watson [28], and secured with stainless steel screws and dental cement. After surgery, the rats were housed in individual cages with food and water *ad libitum *for 10 days when the microdialysis experiment was performed.

### Microdialysis studies

Concentric microdialysis probes were constructed from regenerated cellulose dialysis tubing (MWCO 6000 Da, 225 mm o.d., 2 mm active dialysis length) and fused-silica capillary tubing, the body of the probe being made of a 3 cm 26 G stainless steel tube, which was glued on a flat probe holder (Harvard, USA). Before implantation, the probes were perfused at a rate of 1 μl/min with artificial cerebrospinal fluid (aCSF) (149 mM NaCl, 2.80 mM KCl, 1.2 mM MgCl_2_, 1.2 mM CaCl_2_, 2.78 mM phosphate buffer, pH 7.4). A probe was implanted into the guide-cannula of a freely moving animal placed in the experiment cylinder, the inlet and outlet of the probe being connected to a liquid swivel (mouse model, Instech Solomon USA). The rate of infusion was 1 μl/min for the *in vivo *evaluation of GLU uptake and 2 μl/min for the "no net flux" experiment. For each dialysis experiments, at least 3 hours was allowed to elapse after microdialysis probe implantation before collection of basal samples. At the end of the experiment, Evans blue was injected via the probe, the rats were sacrificed with a lethal dose of pentobarbital, and the placement of the cannula was verified on the frozen brain [29].

#### "No net flux" studies

The basal extracellular concentrations of GLU and GABA were determined by the "no net flux" quantitative microdialysis method [30]. Four different concentrations of GLU (0, 0.5, 1, and 5 μmol/L) or GABA (0, 10, 50, and 100 nanomoles/L) (C_in_), chosen to bracket the expected extracellular concentration, were passed through the microdialysis membrane (each for 16 min, separated by perfusion with aCSF for washing); no sample was collected during the first 10 min of GLU perfusion, allowing a period of equilibration, then three 2-min dialysis samples were collected and stored at -40°C until analyzed for amino acid content.

The concentrations of neurotransmitter in the dialysate samples (C_out_) obtained during perfusion with the various concentrations of neurotransmitter (C_in_) were used to construct a linear regression plot of the net change in neurotransmitter (ΔC = C_in _- C_out_) against C_in _for each animal. The true extracellular levels and the extraction fraction of the probe were determined as described by Parsons and Justice [31]. The true extracellular concentration is equal to C_in _when ΔC = 0 and the extraction fraction (Ed) is determined from the slope of the linear regression. Differences in extracellular concentrations or extraction fractions between GAERS and control rats were tested using the F test followed by either Welch's test or Student's t test, as appropriate.

#### Measurement of amino acid content

On the day of analysis, 4 μL of sample and 4 μL of standard solutions were derivatized at room temperature by adding 1.6 μL of a mixture (1:2:1 v/v/v) of (i) internal standard (10^-4 ^mol/L cysteic acid in 0.117 mol/L perchloric acid), (ii) a borate/NaCN solution (100:20 v/v mixture of 500 mmol/L borate buffer, pH 8.7, and 87 mmol/L NaCN in water), and (iii) a 2.925 mmol/L solution of naphthalene-2,3-dicarboxaldehyde in acetonitrile/water (50:50 v/v). The samples were then analyzed for amino acid content using an automatic capillary zone electrophoresis P/ACE™ MDQ system (Beckman, USA) equipped with a ZETALIF laser-induced fluorescence detector (Picometrics, France). Excitation was performed using a He-Cd laser (Liconix, USA) at a wavelength of 442 nm, the emission wavelength being 490 nm. Separations were carried out on a 63 cm × 50 μm i.d. fused-silica capillary (Composite Metal Services, Worcester, England) with an effective length of 52 cm. Each day, before the analyses, the capillary was sequentially flushed with 0.25 mol/L NaOH (15 min), ultra-pure water (15 min), and running buffer (75 mmol/L sodium borate, pH 9.20 ± 0.02, containing 10 mmol/L HP-β-CD and 70 mmol/L SDS) (5 min). The separation conditions were an applied voltage of 25 kV, hydrodynamic sample injectionOK?? (10 s at 0.6 psi), and a temperature, between 36 and 38°C. The capillary was sequentially flushed for 30 s each with 0.25 mol/L NaOH, ultra-pure water, and running buffer between analyses. Electropherograms were acquired at 15 Hz using P/ACE™ MDQ software [32].

#### Measurements of in vivo GLU uptake

A method based on the uptake of exogenous radioactive GLU [33] was used to evaluate GLU uptake in the cortex and thalamus. Uptake experiments were performed by switching the microdialysis probe perfusion fluid for 10 min to aCSF containing 250 μM ^3^H-GLU (15 Ci/mMol) and 120 μM ^14^C-mannitol (MAN) (60 mCi/mMol) (PerkinElmer Life and Analytical Sciences USA), samples of microdialysis probe effluent being collected every 2 min for dual label scintillation counting. After perfusion with aCSF for 12 min to flush out the radioactive isotopes, the experiment was repeated in the presence of 20 mM threo-β-hydroxyaspartate (THA), a GLU uptake inhibitor.

The relative GLU uptake was determined using the "recovery" vs. "time" curves for 3H-GLU and 14C-MAN, used as the reference, as it is not taken up by cells. The recovery is the counts exiting the probe/input counts ratio (Eq. 1 and 2). The cellular extraction fraction for GLU corrected for mannitol is calculated as in equation 3

R_MAN _= MAN outlet/MAN inlet

R_GLU _= GLU outlet/GLU inlet

E_L-GLU _= R_MAN _- R_GLU_/R_MAN_

The relative GLU uptake in the presence or absence of THA was calculated for each 2 min of the 10 minute infusion period. Differences in uptake between the mean ± SEM for GAERS (N = 12) and NEC rats (N = 12) were tested using the unpaired Student's t test and a level of significance of p < 0.05.

### Western blot analysis

The experiments were performed on adult GAERS (n = 6) and NEC rats (n = 6). Dissection of the frontoparietal cortex and thalamus, brain sample preparation, and electrophoresis were performed as described previously (5). Western blotting was performed using 2, 5, or 7.5 μg of protein per lane for VGLUT1, VGLUT2, or synaptophysin, respectively, and antibodies against VGLUT1 (1/5000) or VGLUT2 (1/1000) (both from Chemicon, UK) or synaptophysin (1/500) (Boehringer, Mannheim, Germany). Bound antibody was detected using enzyme catalyzed fluorescence (ECF Western blotting reagent packs; Amersham Pharmacia Biotech, UK) and visualized using a Fluoro-imager (Molecular Dynamics). Fluorescence was quantified using Image Quant (Molecular Dynamics). Linearity of the relationship between the emitted fluorescence and the protein concentration was verified. The results were then expressed as a percentage of control levels (mean ± SEM) and the data analyzed for statistical significance using the unpaired Student's t test and a level of significance of p < 0.05.

## Authors' contributions

MT, MDB, and SP designed and analyzed the experiments. MT and SP carried out the experiments. MT, MDB, LD, and MFB drafted the manuscript. All authors read and approved the final manuscript.

## References

[B1] Marescaux C, Vergnes M, Depaulis A (1992). Genetic absence epilepsy in rats from Strasbourg – a review. J Neural Transm Suppl.

[B2] Danober L, Deransart C, Depaulis A, Vergnes M, Marescaux C (1998). Pathophysiological mechanisms of genetic absence epilepsy in the rat. Prog Neurobiol.

[B3] Richards DA, Lemos T, Whitton PS, Bowery NG (1995). Extracellular GABA in the ventrolateral thalamus of rats exhibiting spontaneous absence epilepsy: a microdialysis study. J Neurochem.

[B4] Sutch RJ, Davies CC, Bowery NG (1999). GABA release and uptake measured in crude synaptosomes from Genetic Absence Epilepsy Rats from Strasbourg (GAERS). Neurochem Int.

[B5] Dutuit M, Didier-Bazes M, Vergnes M, Mutin M, Conjard A, Akaoka H, Belin MF, Touret M (2000). Specific alteration in the expression of glial fibrillary acidic protein, glutamate dehydrogenase, and glutamine synthetase in rats with genetic absence epilepsy. Glia.

[B6] Dutuit M, Touret M, Szymocha R, Nehlig A, Belin MF, Didier-Bazes M (2002). Decreased expression of glutamate transporters in genetic absence epilepsy rats before seizure occurrence. J Neurochem.

[B7] Pumain R, Louvel J, Gastard M, Kurcewicz I, Vergnes M (1992). Responses to N-methyl-D-aspartate are enhanced in rats with petit mal-like seizures. J Neural Transm Suppl.

[B8] Koerner C, Danober L, Boehrer A, Marescaux C, Vergnes M (1996). Thalamic NMDA transmission in a genetic model of absence epilepsy in rats. Epilepsy Res.

[B9] Dufour F, Nalecz KA, Nalecz MJ, Nehlig A (2001). Modulation of absence seizures by branched-chain amino acids: correlation with brain amino acid concentrations. Neurosci Res.

[B10] Bellocchio EE, Reimer RJ, Fremeau RT, Edwards RH (2000). Uptake of glutamate into synaptic vesicles by an inorganic phosphate transporter. Science.

[B11] Janz R, Sudhof TC, Hammer RE, Unni V, Siegelbaum SA, Bolshakov VY (1999). Essential roles in synaptic plasticity for synaptogyrin I and synaptophysin I. Neuron.

[B12] Hinz B, Becher A, Mitter D, Schulze K, Heinemann U, Draguhn A, Ahnert-Hilger G (2001). Activity-dependent changes of the presynaptic synaptophysin-synaptobrevin complex in adult rat brain. Eur J Cell Biol.

[B13] Pinault D (2003). Cellular interactions in the rat somatosensory thalamocortical system during normal and epileptic 5–9 Hz. J Physiol.

[B14] Meeren H, van Luijtelaar G, Lopes da Silva F, Coenen A (2005). Evolving concepts on the pathophysiology of absence seizures: the cortical focus theory. Arch Neurol.

[B15] Manning JP, Richards DA, Leresche N, Crunelli V, Bowery NG (2004). Cortical-area specific block of genetically determined absence seizures by ethosuximide. Neuroscience.

[B16] Anderson CM, Swanson RA (2000). Astrocyte glutamate transport: review of properties, regulation, and physiological functions. Glia.

[B17] Danbolt NC (2001). Glutamate uptake. Prog Neurobiol.

[B18] Hisano S, Hoshi K, Ikeda Y, Maruyama D, Kanemoto M, Ichijo H, Kojima I, Takeda J, Nogami H (2000). Regional expression of a gene encoding a neuron-specific Na(+)-dependent inorganic phosphate cotransporter (DNPI) in the rat forebrain. Brain Res Mol Brain Res.

[B19] Kaneko T, Fujiyama F, Hioki H (2002). Immunohistochemical localization of candidates for vesicular glutamate transporters in the rat brain. J Comp Neurol.

[B20] Varoqui H, Schafer MK, Zhu H, Weihe E, Erickson JD (2002). Identification of the differentiation-associated Na+/PI transporter as a novel vesicular glutamate transporter expressed in a distinct set of glutamatergic synapses. J Neurosci.

[B21] Ni B, Wu X, Yan GM, Wang J, Paul SM (1995). Regional expression and cellular localization of the Na(+)-dependent inorganic phosphate cotransporter of rat brain. J Neurosci.

[B22] Minelli A, Edwards RH, Manzoni T, Conti F (2003). Postnatal development of the glutamate vesicular transporter VGLUT1 in rat cerebral cortex. Brain Res Dev Brain Res.

[B23] Boulland JL, Qureshi T, Seal RP, Rafiki A, Gundersen V, Bergersen LH, Fremeau RT, Edwards RH, Storm-Mathisen J, Chaudhry FA (2004). Expression of the vesicular glutamate transporters during development indicates the widespread corelease of multiple neurotransmitters. J Comp Neurol.

[B24] LaMantia AS (1995). The usual suspects: GABA and glutamate may regulate proliferation in the neocortex. Neuron.

[B25] Cornell-Bell AH, Thomas PG, Smith SJ (1990). The excitatory neurotransmitter glutamate causes filopodia formation in cultured hippocampal astrocytes. Glia.

[B26] Monnerie H, Shashidhara S, Le Roux PD (2003). Effect of excess extracellular glutamate on dendrite growth from cerebral cortical neurons at 3 days in vitro: Involvement of NMDA receptors. J Neurosci Res.

[B27] Karpova AV, Bikbaev AF, Coenen AML, van Luijtelaar G (2005). Morphometric Golgi study of cortical location in WAG/Rij rats: the cortical focus theory. Neurosci Res.

[B28] Paxinos G, Watson C (1998). The rat brain in stereotaxic coordinates, New York.

[B29] Bert L, Favale D, Jego G, Greve P, Guilloux JP, Guiard BP, Gardier AM, Suaud-Chagny MF, Lestage P (2004). Rapid and precise method to locate microdialysis probe implantation in the rodent brain. J Neurosci Methods.

[B30] Justice JB (1993). Quantitative microdialysis of neurotransmitters. J Neurosci Methods.

[B31] Parsons LH, Justice JB (1994). Quantitative approaches to in vivo brain microdialysis. Crit Rev Neurobiol.

[B32] Sauvinet V, Parrot S, Benturquia N, Bravo-Moraton E, Renaud B, Denoroy L (2003). In vivo simultaneous monitoring of gamma-aminobutyric acid, glutamate, and L-aspartate using brain microdialysis and capillary electrophoresis with laser-induced fluorescence detection: Analytical developments and in vitro/in vivo validations. Electrophoresis.

[B33] Alexander GM, Grothusen JR, Gordon SW, Schwartzman RJ (1997). Intracerebral microdialysis study of glutamate reuptake in awake, behaving rats. Brain Res.

